# Mark-Recapture and Mark-Resight Methods for Estimating Abundance with Remote Cameras: A Carnivore Case Study

**DOI:** 10.1371/journal.pone.0123032

**Published:** 2015-03-30

**Authors:** Robert S. Alonso, Brett T. McClintock, Lisa M. Lyren, Erin E. Boydston, Kevin R. Crooks

**Affiliations:** 1 Department of Fish, Wildlife, and Conservation Biology, Colorado State University, Fort Collins, Colorado, United States of America; 2 Western Ecological Research Center, Biological Resources Discipline, United States Geological Survey, Thousand Oaks, California, United States of America; 3 National Marine Mammal Laboratory, National Marine Fisheries Service, National Oceanic and Atmospheric Administration, Seattle, Washington, United States of America; 4 Western Ecological Research Center, Biological Resources Discipline, United States Geological Survey, Carlsbad, California, United States of America; University of Queensland, AUSTRALIA

## Abstract

Abundance estimation of carnivore populations is difficult and has prompted the use of non-invasive detection methods, such as remotely-triggered cameras, to collect data. To analyze photo data, studies focusing on carnivores with unique pelage patterns have utilized a mark-recapture framework and studies of carnivores without unique pelage patterns have used a mark-resight framework. We compared mark-resight and mark-recapture estimation methods to estimate bobcat (*Lynx rufus*) population sizes, which motivated the development of a new "hybrid" mark-resight model as an alternative to traditional methods. We deployed a sampling grid of 30 cameras throughout the urban southern California study area. Additionally, we physically captured and marked a subset of the bobcat population with GPS telemetry collars. Since we could identify individual bobcats with photos of unique pelage patterns and a subset of the population was physically marked, we were able to use traditional mark-recapture and mark-resight methods, as well as the new “hybrid” mark-resight model we developed to estimate bobcat abundance. We recorded 109 bobcat photos during 4,669 camera nights and physically marked 27 bobcats with GPS telemetry collars. Abundance estimates produced by the traditional mark-recapture, traditional mark-resight, and “hybrid” mark-resight methods were similar, however precision differed depending on the models used. Traditional mark-recapture and mark-resight estimates were relatively imprecise with percent confidence interval lengths exceeding 100% of point estimates. Hybrid mark-resight models produced better precision with percent confidence intervals not exceeding 57%. The increased precision of the hybrid mark-resight method stems from utilizing the complete encounter histories of physically marked individuals (including those never detected by a camera trap) and the encounter histories of naturally marked individuals detected at camera traps. This new estimator may be particularly useful for estimating abundance of uniquely identifiable species that are difficult to sample using camera traps alone.

## Introduction

Reliably estimating abundance of carnivores can be difficult because many populations exist in low densities and are wide-ranging, nocturnal, and secretive [1–3]. Consequently, traditional methods of physical capture have been replaced by the use of non-invasive detection techniques such as remotely-triggered cameras, which offer a viable option for assessment of carnivore abundance [3–7]. Specifically, camera traps are low cost, low maintenance, and create minimal disturbance, and photo records provide information on date, time, activity patterns, and individual identification if animals have natural or artificial individually-distinct marks.

Both mark-recapture [8] and mark-resight [9] methods have been used to estimate population numbers from photo data. Under the photographic mark-recapture framework, researchers non-invasively "capture" animals via photograph and identify individuals by their pelt pattern or other natural markings. After first capture by a camera trap, animals are considered "marked" based on unique natural characteristics. Encounter histories for marked individuals (i.e., those photographed at least once) are constructed for a series of recapture occasions from which detection probability and abundance can be estimated. Photographic mark-recapture studies have focused on a variety of felids with unique pelage patterns, including tigers (*Panthera tigris*) [10], ocelots (*Leopardus pardalis*) [11,12], jaguars (*Panthera onca*) [13], leopards (*Panthera pardus*) [3], snow leopards (*Uncia uncia*) [14], and bobcats (*Lynx rufus*) [15].

Camera data are difficult to use in a mark-recapture framework when animals do not have unique pelage or other natural markings, because individuals cannot be identified by photograph alone. However, if researchers can physically mark some animals and individually identify the tagged animals with photographs, mark-resight models may be appropriate [9,16]. In mark-resight studies, after the initial marking of individuals, there may be one or several resighting occasions in which marked animals are resighted, but unmarked animals remain unmarked and are counted as such. This distinguishes mark-resight from mark-recapture methods because no new marks are introduced during resighting occasions.

Herein we use camera data to estimate population sizes of bobcat within and around an urban coastal reserve in southern California. Due to their sensitivities to urban fragmentation, bobcats have been a focal species in several studies throughout southern California [2,17–20], yet few bobcat density estimates exist for this region (but see Ruell et al. [21]). Bobcats are individually identifiable by pelt patterns, thus photo data for this species can be used with mark-recapture models [15,22]. Additionally, we conducted our camera trap survey in conjunction with an ongoing GPS telemetry study, where animals were physically marked by researchers, thus providing an opportunity to use a mark-resight framework. This unique study design therefore enabled the use of both mark-recapture and mark-resight methods. We compare these approaches and evaluate the potential advantages and disadvantages of each for estimating carnivore abundance using remote cameras. This comparison motivated the development of a new "hybrid" mark-resight model as an alternative to traditional mark-recapture and mark-resight methods.

## Materials & Methods

### Study Area

**Fig 1:**
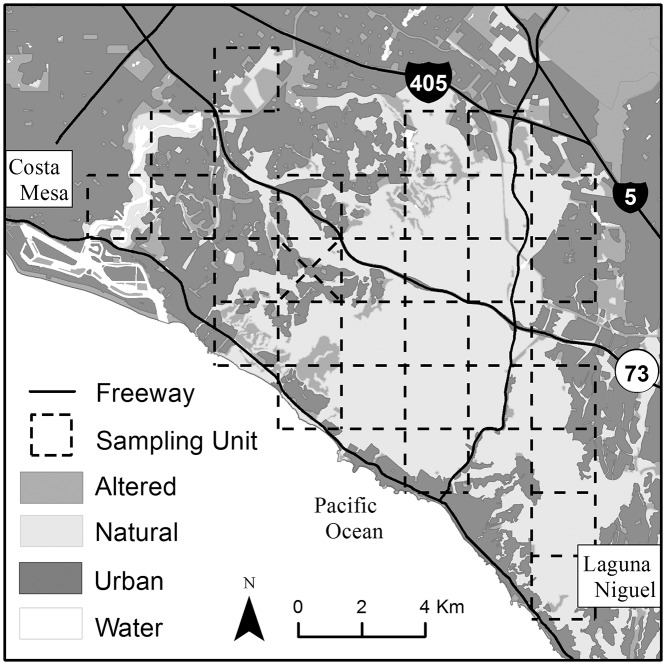
The San Joaquin Hills study area, Orange County, California. The sampling unit grid (dashed lines) was used to determine the locations of remote camera stations. Map figure base layers from SCAG [35] and StreetMap USA for ESRI ArcGIS 9.3, for representational purposes only.

The San Joaquin Hills study area was located within the Coastal Reserve (33°36’N; 117°47’W) of the Nature Reserve of Orange County, south and west of two principal 10-lane freeways between the cities of Costa Mesa and Laguna Niguel, California (Fig 1). The landscape contained a mix of urban and suburban development as well as natural habitat, including undeveloped private property, nature reserves, state parks, and county parks. Natural habitat primarily consisted of coastal sage scrub, chaparral, riparian, coastal oak woodland, and annual grassland communities.

### Camera Trap Survey

Within our study area, we created a square sampling grid consisting of 2 km x 2 km (4 km^2^) sampling units. This unit size represents the average bobcat home range in the nearby North Irvine Ranch [23], a useful approximation for the preferred cell size of camera grids [24] (Fig 1). Given that bobcats typically avoid urbanized areas [19,20], we only considered 30 sampling units that intersected open space parks and reserves for camera monitoring. Each of these sampling units was further subdivided into 16 grid cells measuring 500 m x 500 m each, one of which we randomly selected for installation of a film camera trap (Camtrakker; CamTrak South Inc., Watkinsville, Georgia, USA). We used the presence of bobcat sign (e.g., tracks, scat) and expert opinion to select the specific camera trap locations within each cell to increase the probability of passively detecting bobcats via camera traps along likely movement routes (e.g., dirt trails and roads) within the study area. We attached a single camera to a post and placed it perpendicular to suspected travel routes to capture the best possible photographs for pelt identification and matching [25]. Although a dual camera set up is advantageous in order to photograph both sides of an individual [10,26], funding and logistical constraints precluded this option given the geographic coverage required. We set the cameras with a 3-minute delay between successive photographs, the shortest available delay on the camera model we used, and cameras were set to sample 24 hours a day. We conducted our camera survey from July 2006 to January 2007.

### Physical Capture

In addition to the camera trap surveys, bobcats were detected on the study area via physical captures for a concurrent GPS telemetry research project. Bobcats were captured in cage traps placed in locations based on sign and knowledge of bobcat movement. Bobcats were fitted with a unique combination of an ear tag and colored taping on the GPS telemetry units, or an ear tag and a cat collar after all GPS units were deployed and no longer available. During handling we photographed all animals on both sides of their body in a series of systematic poses to aid in identification of physically captured animals later photographed by camera traps. As described in Heilbrun et al. [25], the poses included photographs of the fore legs, hind legs, torso, face, and tail of the captured animals. These capture photos were compared to the remotely-triggered camera photos to assist with individual identification.

### Ethics statement

We conducted our study under California Department of Fish and Wildlife Scientific Collecting Permit SC 005436, which covered all aspects of the research in the study area, including animal capture and camera traps. Animal Care and Use Committees for the U.S. Geological Survey and Colorado State University (protocol 03 - 187A - 0) approved our study.

### Photograph Identification

We individually identified animals by comparing bobcat photographs using the pelt-pattern identification protocol outlined by Heilbrun et al. [25]. Since our camera traps consisted of a single camera, most useable photos were taken of either the left or right side of the body. Photographs of poor quality due to inadequate lighting, distance (e.g., too close or too far from the camera), and extreme angles (e.g., walking straight into or away from the camera) were not included because individual pelts could not be reliably identified. Following Heilbrun et al. [25], individual bobcats were matched by confirming that at least three natural pelage features (e.g., groupings of leg spots, groupings of body spots, facial markings, and tail markings) or artificial (i.e., human-made) marks (e.g., ear tags or GPS/cat collars) were present in both photographs. The identification of a differing feature between pelt patterns of photographed bobcats indicated unique individuals. Complete encounter history data can be found in McClintock et al. [27].

### Abundance Estimation

#### Mark-recapture

We used closed capture mark-recapture models in program MARK [28] to estimate bobcat abundance. Under the mark-recapture framework, there is an initial capture and marking occasion followed by several recapture occasions where new marks are added to the population [8]. Marks added in either the initial capture occasion or subsequent occasions may be artificial marks (e.g., tags, collars, dyes, etc.) or the identification of natural marks (e.g., individually-unique pelage patterns or other natural characteristics). In our mark-recapture study, we marked animals captured via camera traps by identifying individual pelt patterns. Although bobcats are uniquely identifiable by spot patterns, the patterns are bilaterally asymmetrical [25]. We were therefore unable to match left-side photos with right-side photos because we used a single camera at each station. Consequently, the data were split into left- and right-side encounter histories, and these left- and right-side datasets were analyzed separately. The closed capture mark-recapture models in program MARK require sampling without replacement, meaning individuals are encountered at most once per sampling occasion. We defined a sampling occasion as a 23-day period pooled across all cameras such that capture probabilities were >0.1 [8], yielding a total of 8 sampling occasions during the study period. An individual photographed at least once within any given 23-day sampling occasion was considered captured for that occasion. To explore heterogeneity in detection probability, we examined models that assumed no capture heterogeneity (model Mo), capture heterogeneity due to time (Mt) corresponding to the wet (December-January) and dry (July-November) seasons, capture heterogeneity based on individual sources of variation using two mixtures (Mh2), and capture heterogeneity due to seasons and individual variation (Mth2) [8,29]. We calculated abundance by model averaging over the entire model set for each side-specific estimator [30].

#### Mark-resight

Under the mark-resight framework, an initial marking period is followed by resighting occasions where, unlike the mark-recapture framework, no new marks are added to the population. Thus, unmarked animals remain unmarked throughout the study [9]. In our study, the initial capture occasion occurred during the physical capture and artificial marking of bobcats with a unique combination of ear tags and collars for an ongoing GPS telemetry project. Bobcats that were never physically captured were considered unmarked throughout the study. After the physical capture period, we used the remotely-triggered camera grid to resight both marked and unmarked animals. Because we physically marked animals and documented pelt patterns on both sides during the initial marking period, we were able to combine both the left- and right-side photo datasets for the mark-resight analysis. We used the Poisson log-normal (PNE) mark-resight estimator in program MARK [31] to estimate bobcat abundance. Unlike the mark-recapture models discussed above, sampling may be with replacement for the PNE, so distinct sampling occasions do not need to be delineated; instead, all marked and unmarked individual photo resightings were counted over the duration of the study. For the PNE mark-resight analysis, we examined models with and without the individual heterogeneity parameter (σ). As with the mark-recapture models, we calculated abundance by model averaging over the entire model set for each estimator [30].

#### Hybrid mark-resight

Neither traditional mark-recapture nor mark-resight methods utilize all of the available information about the resighting process afforded by this unique study design. The mark-recapture approach uses encounter histories for all individuals based on pelt patterns, but ignores the number of artificially marked individuals that were known to be alive and never photographed during the study period. The traditional mark-resight approach accounts for artificially marked individuals that were known to be alive but never photographed, thus making it more robust to individual sighting heterogeneity than capture-recapture methods. However, only the encounter histories for individuals that are artificially marked are used, and the encounter histories based on individual pelt patterns must be collapsed into simple counts of unmarked individuals. To take advantage of the information provided by both natural pelt patterns and artificially marked individuals, we developed a "hybrid" Poisson log-normal mark-resight estimator (hPNE) for comparison to the traditional mark-recapture and mark-resight estimators described above.

The hPNE uses a Poisson model for resightings of the *m* artificially-marked individuals and a zero-truncated Poisson model for sightings of the *n* individually-identifiable (but not artificially-marked) individuals photographed at least once. The likelihood for the individual sighting rate parameter, *λ*_*i*_, is therefore

$$L\left(\lambda |y\right)=\left[\overset{m}{\underset{i=1}{\prod }}\frac{\lambda_{i}^{y_{i}}\exp\left(-\lambda_{i}\right)}{y_{i}!}\right]\left[\overset{m+n}{\underset{i=m+1}{\prod }}\frac{\lambda_{i}^{y_{i}}\exp\left(-\lambda_{i}\right)}{y_{i}!\left(1-\exp\left(-\lambda_{i}\right)\right)}\right]$$ (1)

where *λ*_*i*_ *=* exp(*α*_*i*_), and *y*_*i*_ is the number of times individual *i* = 1,...,*m*+*n* was photographed during the study period. Here, *y*_*i*_ ϵ {0,1,…}for *i* = 1,...,*m*, and *y*_*i*_ ϵ {1,2,…}for *i* = *m*+1,...,*m*+*n*. To accommodate individual heterogeneity in sighting rates, we assume *α*_*i*_ *~ N*(*μ*,*σ* ^2^), where *α*_*i*_ *=* log(*λ*_*i*_). We further assume *n*|*N*,p*~Binomial(*N*-*m*,p*), where

$$p^{*}=1-\overset{\infty }{\underset{-\infty }{\int }}\exp\left(-\exp\left(\alpha \right)\right)N\left(\alpha ;\mu ,\sigma^{2}\right)d\alpha$$

is the mean probability of being photographed at least once, and *N(α*;*μ*,*σ* ^2^)is the normal density. This model can be easily implemented using Bayesian analysis methods, and we completed our specification with the priors *N*∝ 1/*N*, *μ*~*N*(0,1), and *σ* ^2^~Γ^-1^(3,2)

We performed separate hPNE analyses for the left-side and right-side datasets. In both analyses, we compared models with and without individual heterogeneity (i.e., *σ* ^2^ = 0) using the Deviance Information Criterion (DIC, [32]). For each model, 400000 samples were drawn from the posterior distribution (after burn in) using OpenBUGS ([33]; see S1 Appendix for BUGS code). For each model, we ran three chains initiated from overdispersed starting values and found no evidence for lack of convergence based on standard diagnostics implemented in OpenBUGS.

### Density Estimation

**Fig 2:**
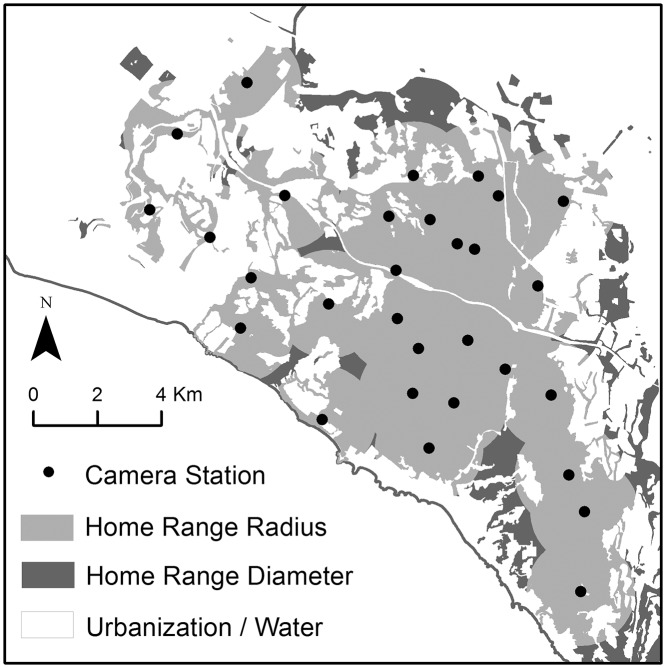
The effective study area using the radius and diameter of mean bobcat home range size (8.83 km^2^) buffered around camera stations. Urban or water land use classifications [35] were removed from the buffers. Map figure base layer from SCAG [35] for representational purposes only.

For comparison with other bobcat population studies, we calculated population density from each abundance estimate. Density estimates can be sensitive to methods used to determine the size of a study area [3,12]. As such, it may be difficult to compare densities if methods of study area delineation vary across studies [12]. To determine the size of the effective study area, we used methods similar to Ruell et al. [21], who estimated bobcat densities via non-invasive fecal DNA surveys in the Santa Monica Mountains north of Los Angeles. We used our GPS telemetry data to calculate mean bobcat home range size (8.83 km^2^, 95% CI 5.26–12.39 km^2^, n = 8 males and 6 females), estimated with a fixed 95% kernel [34]. We then created buffers in ArcGIS 9.3 (ESRI, Redlands, California, USA) around each camera location for the radius (1.68 km, 95% CI 1.29–1.99 km) and the diameter (3.35 km, 95%CI 2.59–3.97 km) of the mean home range size. The buffers were dissolved into one layer for the radius and one layer for the diameter measurements, thus eliminating overlap between the individual camera buffers. Following Ruell et al. [21], we removed areas of intense urbanization from the buffer, which our GPS-collared bobcats generally avoided; 7% (SE = 2%) of GPS locations per individual were located in urban areas, as classified by GIS land-use layers from the Southern California Association of Governments [35]. The buffer did include golf courses, regional parks, riparian strips, and natural habitat, which bobcats used more frequently (Fig 2).

Following Ruell et al. [21], to calculate density point estimates we divided our abundance estimate by the effective study area derived from the mean home range radius and again separately by the area derived from the mean home range diameter (see Table 1). Standard errors were approximated using the delta method. Confidence intervals for abundance and density were calculated as 95% logarithm-transformed normal for the mark-recapture and PNE models. For hPNE, we calculated 95% highest posterior density intervals for abundance and density.

**Table 1:** Camera survey model-averaged mark-recapture and mark-resight bobcat *Lynx rufus* abundance ($\hat{N}$) and density/km^2^ ($\hat{D}$) estimates in the San Joaquin Hills study area, Orange County, California.

Estimator	*$\hat{N}$*	95% CI	*${\hat{D}}_{r}$*	95% CI	*${\hat{D}}_{d}$*	95% CI	% CIL
Mark-recapture (RS)	44	30–87	0.45	0.31–0.90	0.37	0.25–0.73	131
Mark-recapture (LS)	36	27–66	0.37	0.28–0.68	0.30	0.23–0.55	107
Mark-resight	56	39–97	0.58	0.40–1.01	0.47	0.33–0.82	104
Hybrid mark-resight (RS)	55	43–70	0.57	0.46–0.74	0.46	0.36–0.59	49
Hybrid mark-resight (LS)	60	45–79	0.62	0.48–0.83	0.50	0.38–0.66	57

Right-side (RS) and left-side (LS) analyses were conducted for the mark-recapture and hybrid mark-resight estimators. Separate density estimates were derived from the estimated radius (${\hat{D}}_{r}$) and diameter (${\hat{D}}_{d}$) of average home range size. % CIL denotes the 95% confidence (or highest posterior density) interval length relative to $\hat{N}$ and $\hat{D}$.

### Assumptions

Closed population abundance estimation, including both mark-recapture and mark-resight estimators, entails 3 key assumptions: 1) the study population is closed both geographically and demographically; 2) marks (both natural and artificial) are not lost; and 3) marks are properly identified [8,16]. We believe we satisfied the assumption of geographic closure because urbanization surrounded the study area to the north, east, and south, while the Pacific Ocean bordered the study area to the west. In addition, we did not detect any bobcats moving onto or off of the study area via GPS telemetry or remotely-triggered cameras during the study. Kittens are typically born in the spring and early summer, and we did not document any births during the study period (July to January). We therefore suspected few (if any) births during the study and were not particularly concerned about violations of demographic closure due to reproduction. However, the demographic closure assumption was not met because we documented bobcat mortality during the study period, including four bobcat road kill mortalities. Nevertheless, we found little evidence for closure violations based on open population models for our left- and right-sided mark-recapture data (see S2 Appendix). We note that when demographic closure is violated due to mortality or permanent emigration, the mark-resight models provide an estimate of the population at the beginning of the study period.

Regarding the second and third assumptions, we observed retention of the artificial marks, and the bobcat pelt patterns used to identify individuals do not change over time [22,25]. In addition, the pelt pattern identification protocol outlined by Heilbrun et al. [25] was designed to minimize incorrect matching of bobcat pelts by requiring that the three pelage features on the animals (or one artificial mark) must match. Identifying at least one differing feature between animals confirmed unique individuals. Several researchers in this study independently reviewed and confirmed the matches.

The mark-resight estimators have one additional assumption that marked animals and unmarked animals have independently and identically distributed resighting probabilities. This assumption requires that the marked sample accurately represents the sighting probabilities of the entire population and that sighting probability is independent of mark status [36]. We believe this assumption was reasonably satisfied by using a different method and site locations for physical capture and marking (i.e., cage traps) from the resighting method (i.e., camera traps).

Finally, the models require that the number of physically-marked animals in the population be known, and we determined this from the known bobcats at the beginning of the camera survey via GPS collaring efforts and additional camera sampling (from other ongoing projects) of physically-marked individuals.

## Results

We captured 109 bobcat photos in 4,669 camera trap nights (number of nights that camera traps were operating). Seventeen of those photos were not used in the mark-recapture or mark-resight analysis due to poor photo quality. For the closed capture mark-recapture analysis, we organized the usable photographs into two datasets consisting of 49 right- and 42 left-side photos. Since sampling is without replacement for the closed capture mark-recapture estimator, we were able to use only 35 right-side photos and 34 left-side photos because some individuals were captured multiple times within a 23-day sampling occasion. We identified 23 individual bobcats in the left-side photo data set (16 captured once, 5 captured twice, 1 captured thrice, and 1 captured four times) and 23 individuals in the right-side photo data set (17 captured once, 3 captured twice, 2 captured thrice, and 1 captured six times). Based on physical and photo captures, the minimum count of individuals known to be in the population during our study was 41 bobcats.

Model-averaged mark-recapture point estimates slightly differed between the right-side (44 bobcats) and left-side (36 bobcats) datasets, but they were not significantly different given overlapping 95% confidence intervals (Table 1). The right- and left-side datasets supported differing models with respect to capture heterogeneity (Table A in S3 Appendix). The right-side dataset most supported the model with an individual heterogeneity effect, as well as that with an individual heterogeneity effect and a seasonal time effect. The left-side dataset most supported the null model with no capture heterogeneity or seasonal time effects. The model with a seasonal time effect received some support, but models including individual capture heterogeneity had little support.

Under the PNE mark-resight framework, sampling is with replacement, so all usable left- and right-side photographs were combined into one dataset. We physically captured and marked *m* = 27 bobcats on the study area, and 15 of those animals were resighted with the remotely-triggered camera grid, resulting in 45 photographs of marked individuals and 47 photographs of unmarked individuals. For this analysis, we were able to use one additional photograph known to contain a marked individual, but not identified to individual identity [31]. The PNE analysis showed strong support for the individual heterogeneity model (Table B in S3 Appendix) and the abundance estimate of 56 bobcats was higher than the mark-recapture abundance estimates, but not significantly different (Table 1).

For the hPNE mark-resight framework, sampling is with replacement for the left- and right-side datasets. The right-side analysis consisted of 49 individual photographs, with 26 photos of 10 resighted animals for the *m* = 27 artificially marked individuals and 23 photos of the *n* = 13 naturally marked individuals. The left-side analysis consisted of 42 individual photographs, with 18 photos of 9 resighted animals for the *m* = 27 artificially marked individuals and 24 photos of the *n* = 14 naturally marked individuals. In both analyses, DIC indicated strong support for the individual heterogeneity models (Table C in S3 Appendix). The hPNE point estimates for the right-side (55 bobcats) and left-side (60 bobcats) datasets were similar to the other estimators, but precision was markedly improved (Table 1). Percent confidence interval lengths for the traditional mark-recapture and mark-resight estimators all exceeded 100%, but the percent highest posterior density interval lengths for hPNE were 49% and 57% for the right- and left-side analyses, respectively.

The effective study area size ranged from 96.2 km^2^ derived from the radius of the average bobcat home range, to 119.1 km^2^ derived from the diameter of the average home range. Density point estimates ranged from 0.37–0.62 and 0.30–0.50 bobcats per km^2^ with the study areas defined by the radius and diameter of an average bobcat home range, respectively (Table 1).

## Discussion

The unique design of our remote camera study with an ongoing GPS telemetry study enabled us to compare the use of mark-resight and mark-recapture frameworks to estimate the abundance and density of a fragmentation-sensitive carnivore, the bobcat in urban southern California. Although the datasets were different with respect to how natural marks and artificial marks were handled, or whether sampling was allowed to be with or without replacement during the study period, we found traditional mark-recapture and PNE mark-resight estimators performed similarly. However, because the hybrid mark-resight estimator (hPNE) utilized the most information from the data, it outperformed the other estimators with respect to precision.

Our estimates of bobcat density from hPNE (0.46 to 0.62 per km^2^) were slightly higher than estimates for other study areas in the region (0.25 to 0.42 bobcats/km^2^) using non-invasive scat survey mark-recapture methods [21]. Ruell et al. [21] suggested that relatively low densities could be due to a recent notoedric mange epizootic in their bobcat population, and that previous densities in the area were suspected to be ≥ 0.6 km^2^ as estimated from radio-telemetry data. This suggests that the densities we estimated from our camera trap study area were reasonable and within the bounds of similar density estimates for bobcats in the region generated with other approaches.

In a non-invasive remotely-triggered camera study, both the mark-resight and the mark-recapture frameworks present advantages and disadvantages. If animals can be individually identified via natural pelage markings, the mark-recapture framework presents a clear advantage because animals may never need to be physically handled. In our study, however, we were limited to a single camera at each station and thus needed to split our data because we could not reliably match right- and left-side photos due to asymmetrical pelt patterns; this resulted in lower sample sizes and decreased precision. Another drawback of separate left- and right-sided analyses is that they result in two abundance estimates for the same population. In practice, a single estimate is typically preferred for management, and there is no single "correct" method for obtaining one. The simplest options include a variance-weighted average of the left- and right-sided abundance estimates or selecting the estimate with the highest precision. A recently developed statistical method now allows integrated mark-recapture analyses with bilateral photo-identification records [27,37], but we did not utilize these models because they have yet to be extended to allow for individual variation in parameters (but see [38]). A dual camera trap setup [10,26] would have enabled matching of right- and left-side photos, likely increasing capture probability and precision. Additionally, increasing the density of camera traps on the landscape, and including multiple trap sites per smallest female home range [39,40], would help achieve greater precision.

In comparison, under the mark-resight framework, animals need to be marked prior to conducting independent resighting surveys. If the premarking involves physical capture and artificial marking, as was the case in our study, it is typically more invasive and costly than the mark-recapture framework if all animals can be individually identified via natural markings. However, if researchers are already handling animals, for example to deploy telemetry collars, then the concurrent use of remote cameras to acquire non-invasive resightings presents a viable opportunity for the use of the mark-resight framework for abundance estimation. When complete resighting histories are known for the artificially marked subset of the population, including those never photographed by camera traps, mark-resight methods are more robust to individual sighting heterogeneity than traditional mark-recapture. Unmodeled individual heterogeneity will often result in underestimation of abundance [8,31]. The minimum number of individuals known to be alive during our study was 41 bobcats, and the lower bounds for the mark-recapture estimates could be indicative of bias induced by unmodeled individual heterogeneity.

The mark-resight framework offers other benefits. First, the ability to use one camera to detect artificial marks in a mark-resight study, as opposed to two cameras to detect bilaterally asymmetrical natural markings in a mark-recapture study, is another potential advantage of the mark-resight framework. The process of matching marks (natural or artificial) among photos may also be less intensive, because only those animals who were captured and marked in the initial capture session need to be identified, as opposed to the mark-recapture estimators that require individual identification of every photograph. Finally, sampling can be with replacement for the PNE and hPNE mark-resight estimators, so delineation of secondary sampling occasions is not necessary. This is natural for camera trap data; generally cameras traps are placed on the landscape and run for the duration of the study period, with no accounting for distinct sampling occasions without replacement. Sampling with replacement allows all identifiable photographs to be used, a contrast with the mark-recapture estimators, in which multiple detections of the same individual within the same sampling occasion are discarded (i.e., only one detection of an individual within a sampling occasion is used by the estimator).

As was demonstrated by the hPNE mark-resight results, we found there may be notable advantages to conducting an independent survey (e.g., via physical trapping) prior to initiating camera trap surveys, even when all individuals in the population are naturally marked. By having a subset of the population for which complete encounter histories are known, including those never photographed by a camera trap, precision can be greatly improved and bias due to individual sighting heterogeneity reduced. In our case study, we found that traditional mark-recapture and mark-resight methods produced insufficient sample sizes to achieve desirable levels of precision. However, when integrated using the new hPNE model, the information afforded by the artificially-marked individuals and natural pelt patterns of individuals without artificial marks achieved a desirable level of precision. When all individuals in a population are naturally marked and it is difficult to achieve sufficient sample sizes from camera surveys alone (e.g., due to the rarity or elusiveness of the focal species), then investment in an independent non-camera survey may provide critical information that will improve inference about population size.

Finally, spatial capture-recapture methods (e.g., [41,42]) are becoming increasingly popular for the analysis of felid camera-trap data. While analogous mark-resight methods were undeveloped at the time of this research, spatial mark-resight models have since started to emerge [43,44]. These approaches are quickly becoming the standard for estimating the density of rare and elusive carnivores, and they alleviate the need for *ad-hoc* effective study area calculations for density estimation (such as those used in this paper) [45]. While many of our findings are relevant to both non-spatial and spatially-explicit approaches, a similar comparison of spatial mark-recapture and spatial mark-resight methods remains a promising avenue for future research.

## Supporting Information

S1 Appendix BUGS model specification code for implementing the hybrid Poisson log-normal mark-resight abundance estimator (hPNE). This file also contains Figure A and Figure B. Models include individual sighting heterogeneity (Figure A) or no individual sighting heterogeneity (Figure B).
(DOCX)

S2 Appendix This file contains Table A and Table B. Table A, Examination of closure assumption for mark-recapture analysis using left-sided data. Table B, Examination of closure assumption for mark-recapture analysis using right-sided data.
(DOCX)

S3 Appendix This file contains Tables A-C. Table A, Individual model results for the mark-recapture analysis. Table B, Individual model results for the mark-resight analysis. Table C, Individual model results for the hybrid mark-resight analysis.
(DOCX)
