# Supplementary material for: Mark-Recapture and Mark-Resight Methods for Estimating Abundance with Remote Cameras: A Carnivore Case Study
Source: PLoS One. 2015 Mar 30;10(3):e0123032. doi: 10.1371/journal.pone.0123032 (PMC4378916; doi:10.1371/journal.pone.0123032)
Supplement: S1 Appendix — This file also contains Figure A and Figure B. Models include individual sighting heterogeneity (Figure A) or no individual sighting heterogeneity (Figure B). (DOCX) [file pone.0123032.s001.docx]

**S1 Appendix.** BUGS model specification code for implementing the hybrid Poisson log-normal mark-resight abundance estimator (hPNE). Models include individual sighting heterogeneity (Figure A) or no individual sighting heterogeneity (Figure B).

**Figure A. BUGS code for hPNE with individual heterogeneity.**

model

{

for(i in 1:n) {

alpha[i] ~ dnorm(mu,tau)

lambda[i] <- exp(alpha[i])

dummy[i] <- 0

dummy[i]~dloglik(logLike[i])

logLike[i] <- y[i]*log(lambda[i])-lambda[i]

-logfact(y[i])-log(1-exp(-lambda[i]))

}

for(j in (n+1):M) {

alpha[j] ~ dnorm(mu,tau)

lambda[j] <- exp(alpha[j])

y[j] ~ dpois(lambda[j])

}

mu~dnorm(0,1)

tau~dgamma(3,2)

sigma2 <- 1/tau

sigma <- sqrt(sigma2)

F(nu) <- exp(-exp(mu+sigma*nu))*1/sqrt(2*Pi)*exp(-pow(nu,2)/2)

Pi <- 3.14159265359

oneminuspstar <- integral(F(nu), -20, 20, 1.0E-7)

pstar <- 1 - oneminuspstar

# Scale prior for N over {n,n+1,...,M}

n00 ~ dcat(prior[]) # prior for N is {M,...,supN}

N <- n00 - 1 + M

# Use zero trick

dummytoo <- 0

dummytoo ~ dloglik(logzero)

logzero <- loggam(N-(M-n)) - loggam(N-M+1) - loggam(n+1)

+ n*log(pstar) + (N-M)*log(1-pstar)

}

**Figure B. BUGS code for hPNE with no individual heterogeneity.**

model

{

alpha ~ dnorm(0,1)

mu <- exp(alpha)

for(i in 1:n) {

dummy[i] <- 0

dummy[i] ~ dloglik(logLike[i])

logLike[i] <- y[i]*log(mu)-mu-logfact(y[i])-log(1-exp(-mu))

}

for(j in (n+1):M) {

y[j] ~ dpois(mu)

}

pstar <- 1 - exp(-mu)

# Scale prior for N over {n,n+1,...,M}

n00 ~ dcat(prior[])

N <- n00 - 1 + M

# Use zero trick

dummytoo <- 0

dummytoo ~ dloglik(logzero)

logzero <- loggam(N-(M-n)) - loggam(N-M+1) - loggam(n+1)

+ n*log(pstar) + (N-M)*log(1-pstar)

}
