# Supplementary material for: Mark-Recapture and Mark-Resight Methods for Estimating Abundance with Remote Cameras: A Carnivore Case Study
Source: PLoS One. 2015 Mar 30;10(3):e0123032. doi: 10.1371/journal.pone.0123032 (PMC4378916; doi:10.1371/journal.pone.0123032)
Supplement: S2 Appendix — Table A, Examination of closure assumption for mark-recapture analysis using left-sided data. Table B, Examination of closure assumption for mark-recapture analysis using right-sided data. (DOCX) [file pone.0123032.s002.docx]

**S2 Appendix.** Examination of closure assumption for mark-recapture analysis using left-sided (Table A) and right-sided (Table B) data.

We investigated the closure assumption for our mark-recapture data using the Link and Barker [[1](#_ENREF_1)] parameterization of the Jolly-Seber model implemented in Program MARK [[2](#_ENREF_2)]. This parameterization is in terms of detection probability ($p$), apparent survival (), and recruitment rate (). Using AIC_c_, we compared open models (i.e., and/or ) with closed models (i.e., and ). We used the most general temporal model for *p* while allowing constant or temporal models for and . For both the left- and right-sided data, we found weak evidence for violations of closure due to recruitment (birth/immigration) or mortality/permanent emigration (Tables A and B).

**References**

1. Link WA, Barker RJ (2005) Modeling association among demographic parameters in analysis of open population capture-recapture data. Biometrics 61: 46-54.

2. White GC, Burnham KP (1999) Program MARK: survival estimation from populations of marked animals. Bird Study 46 Supplement: S120-S139.

**Table A. AIC_c_ model selection results for open population models using the left-sided mark-recapture data.**

| **Model** | **Delta AIC_c_** | **AIC_c_ weight** | **Num. Parameters** |
| --- | --- | --- | --- |
|  | 0.00 | 0.62 | 8 |
|  | 2.18 | 0.21 | 9 |
|  | 3.35 | 0.12 | 9 |
|  | 4.64 | 0.06 | 10 |
|  | 27.06 | 0.00 | 15 |
|  | 31.54 | 0.00 | 15 |
|  | 96.62 | 0.00 | 22 |

For open population models (i.e., or ), constant (.) and temporal (*t*) effects were included for or . All models include temporal effects on detection probability ().

**Table B. AIC_c_ model selection results for open population models using the right-sided mark-recapture data.**

| **Model** | **Delta AIC_c_** | **AIC_c_ weight** | **Num. Parameters** |
| --- | --- | --- | --- |
|  | 0.00 | 0.67 | 8 |
|  | 2.77 | 0.17 | 9 |
|  | 3.48 | 0.12 | 9 |
|  | 5.64 | 0.04 | 10 |
|  | 32.40 | 0.00 | 15 |
|  | 34.74 | 0.00 | 15 |
|  | 106.42 | 0.00 | 22 |

For open population models (i.e., or ), constant (.) and temporal (*t*) effects were included for or . All models include temporal effects on detection probability ().
