# Supplementary material for: Mark-Recapture and Mark-Resight Methods for Estimating Abundance with Remote Cameras: A Carnivore Case Study
Source: PLoS One. 2015 Mar 30;10(3):e0123032. doi: 10.1371/journal.pone.0123032 (PMC4378916; doi:10.1371/journal.pone.0123032)
Supplement: S3 Appendix — Table A, Individual model results for the mark-recapture analysis. Table B, Individual model results for the mark-resight analysis. Table C, Individual model results for the hybrid mark-resight analysis. (DOCX) [file pone.0123032.s003.docx]

**S3 Appendix.** Individual model results for the mark-recapture (Table A), mark-resight (Table B), and hybrid mark-resight (Table C) analyses.

**Table A. Camera survey closed capture mark-recapture model results for bobcats (*Lynx rufus*) in the San Joaquin Hills study area, Orange County, California.**

| **Model** | **Delta AIC_c_** | | **Model weight** | | **$\hat{N}$** | **SE** | | **95% CI** | |
| --- | --- | --- | --- | --- | --- | --- | --- | --- | --- |
|  |  | |  | |  |  | | **Lower** | **Upper** |
| **Right-side analysis** | |  | |  |  | |  |  |  |
| Mh2 | 0.0 | | 0.49 | | 45 | 13.1 | | 30 | 88 |
| Mth2 | 0.2 | | 0.44 | | 44 | 12.9 | | 30 | 87 |
| Mo | 5.0 | | 0.04 | | 34 | 6.5 | | 27 | 55 |
| Mt | 5.4 | | 0.03 | | 34 | 6.4 | | 27 | 55 |
| **Left-side analysis** | |  | |  |  | |  |  |  |
| Mo | 0.0 | | 0.54 | | 35 | 7.3 | | 27 | 59 |
| Mt | 1.0 | | 0.33 | | 35 | 7.2 | | 27 | 59 |
| Mh2 | 3.8 | | 0.08 | | 40 | 14.2 | | 27 | 94 |
| Mth2 | 4.9 | | 0.05 | | 40 | 14.1 | | 27 | 93 |

Capture heterogeneity models include no heterogeneity (Mo), seasonal heterogeneity (Mt), individual heterogeneity with 2 mixtures (Mh2), and both seasonal and individual heterogeneity with 2 mixtures (Mth2).

**Table B. Camera survey mark-resight (PNE) model results for bobcats (*Lynx rufus*) in the San Joaquin Hills study area, Orange County, California.**

| **Model** | **Delta AIC_c_** | **Model weight** | **$\hat{N}$** | **SE** | **95% CI** | |
| --- | --- | --- | --- | --- | --- | --- |
|  |  |  |  |  | **Lower** | **Upper** |
| α(.) σ(.) | 0.00 | 1.00 | 56 | 13.8 | 39 | 97 |
| α(.) σ(0) | 32.67 | 0.00 | 55 | 5.8 | 46 | 69 |

Covariate models on intercept (α) and individual heterogeneity (σ) parameters include intercept only (.) and no effect (0).

**Table C. Camera survey hybrid mark-resight (hPNE) model results for bobcats (*Lynx rufus*) in the San Joaquin Hills study area, Orange County, California.**

| **Model** | **Delta DIC** | **$\hat{N}$** | **SE** | **95% HPDI** | |
| --- | --- | --- | --- | --- | --- |
|  |  |  |  | **Lower** | **Upper** |
| **Right-side analysis** |  |  |  |  |  |
| α(.) σ(.) | 0 | 55 | 7.6 | 43 | 70 |
| α(.) σ(0) | 24.9 | 47 | 4.0 | 41 | 55 |
| **Left-side analysis** |  |  |  |  |  |
| α(.) σ(.) | 0 | 60 | 9,4 | 45 | 79 |
| α(.) σ(0) | 6.5 | 52 | 5.7 | 43 | 64 |

Covariate models on intercept (α) and individual heterogeneity (σ) parameters include intercept only (.) and no effect (0).
